# Electroconvulsive therapy in an adolescent with dissociative identity disorder and depression: a case report

**DOI:** 10.3389/fpsyt.2026.1811215

**Published:** 2026-04-16

**Authors:** Lipeng Zhu, Mengmeng Zhang, Yunsheng Hou, Ruihua Yang, Juan Wang

**Affiliations:** 1 Psychiatry Department, The Second Affiliated Hospital of Henan Medical University, Xinxiang, China; 2 Mental Health and Artificial Intelligence Research Center, The Second Affiliated Hospital of Henan Medical University, Xinxiang, China

**Keywords:** case report, dissociative identity disorder, electroconvulsive therapy, major depressive disorder, suicidal ideation

## Abstract

**Background:**

Dissociative identity disorder (DID) is a psychiatric condition characterized by the existence of at least two distinct identities. This disorder often serves as a defense mechanism, a response to severe childhood trauma, aimed at protecting the individual from overwhelming emotions or experiences. There is no specific targeted treatment for DID at present.

**Objective:**

The purpose of this report is to explore the safety and efficacy of electroconvulsive therapy (ECT) in combination with pharmacological treatment for the treatment of concomitant depression in an adolescent female with DID, and to provide new clinical ideas and empirical evidence for similar cases.

**Case summary:**

A 15-year-old adolescent female presented with three distinct personality states, accompanied by hallucinations, negative thoughts, and self-harming behaviors. She was diagnosed with dissociative identity disorder and depression according to DSM-5 criteria. Following combined ECT and medication treatment, the patient’s disparate personalities were integrated, and depressive symptoms were alleviated. During ECT administration, no significant adverse reactions occurred beyond mild headaches and transient memory impairment.

**Conclusion:**

For adolescent patients with DID comorbid with depression who are at high risk of suicide, ECT combined with pharmacological treatment may be considered an effective and relatively safe treatment strategy in emergency situations. It is likely that the combined use of ECT, medication and psychotherapy has led to an improvement in the patient’s condition.

## 1 Introduction

Dissociative Identity Disorder (DID) is a complex and controversial psychiatric disorder characterized by the presence of two or more distinct identities, personality states, or identities that repeatedly control an individual’s behavior (1). Reports have indicated that the prevalence of DID is relatively high, at approximately 6% among psychiatric outpatients (2), and 1–3% in the general population (3).

The etiology of DID remains unclear. The trauma model states that DID is a severe form of Post-Traumatic Stress Disorder (PTSD) stemming from severe and prolonged trauma (4). The fantasy model postulates that DID is primarily caused by suggestion and playacting and is exacerbated by a high propensity for fantasy and susceptibility to suggestion (4). The social cognitive model suggests that personality alterations are socio-cultural constructs, unrelated to trauma, due to attention-seeking behaviors rather than deception, and unrelated to conscious effort (5, 6). The evidence consistently supports the trauma model of DID (7–9), which is associated with early childhood psychological trauma, including factors such as disrupted attachment relationships, prolonged neglect, and abuse (8).

In clinical practice, DID is rarely diagnosed. The core symptoms of DID (e.g., identity alteration, dissociative amnesia) are insidious and often overlooked by patients due to the non-specificity of the symptoms. Symptomatic episodes are context-dependent and typical manifestations may be difficult to observe in non-stressful situations (10). Patients with DID experienced an average of 2.8 misdiagnoses (11) before receiving a correct diagnosis, and it takes 6.7-8 years from first contact with the health care system to a correct diagnosis (11, 12). The most commonly misdiagnosed psychiatric disorders were for affective disorders (63.7%), personality disorders (57.4%), anxiety disorders (44.3%), and schizophrenia (40.8%) (12).

DID is often complicated by comorbidity with major depressive disorder (MDD). Patients often present with persistent symptoms such as depressed mood and diminished interest. Some may even experience suicidal ideation or behaviors. Studies have shown that 61%–72% of DID patients have attempted suicide, which is closely associated with comorbid depression (10). To reduce the risk of self-harm and suicide as quickly as possible, electroconvulsive therapy (ECT) has been used to treat DID with comorbid depression in addition to psychiatric medication (13, 14). The reported patients were adult patients. The ECT appeared to be helpful in relieving dissociative symptoms and depressive symptoms without serious adverse effects. Whether the same therapeutic effect can be achieved in adolescents has not been reported in the literature. This report describes a case of an adolescent female patient with DID complicated by depression, who had severe suicidal ideation and self-harm behaviors. After treatment with ECT combined with psychiatric medication, her clinical symptoms improved.

The purpose of this report is to explore the safety and efficacy of ECT in combination with pharmacological treatment for the treatment of concomitant depression in an adolescent female with DID, and to provide new clinical ideas and empirical evidence for similar cases.

## 2 Case presentation

**Figure 1:**
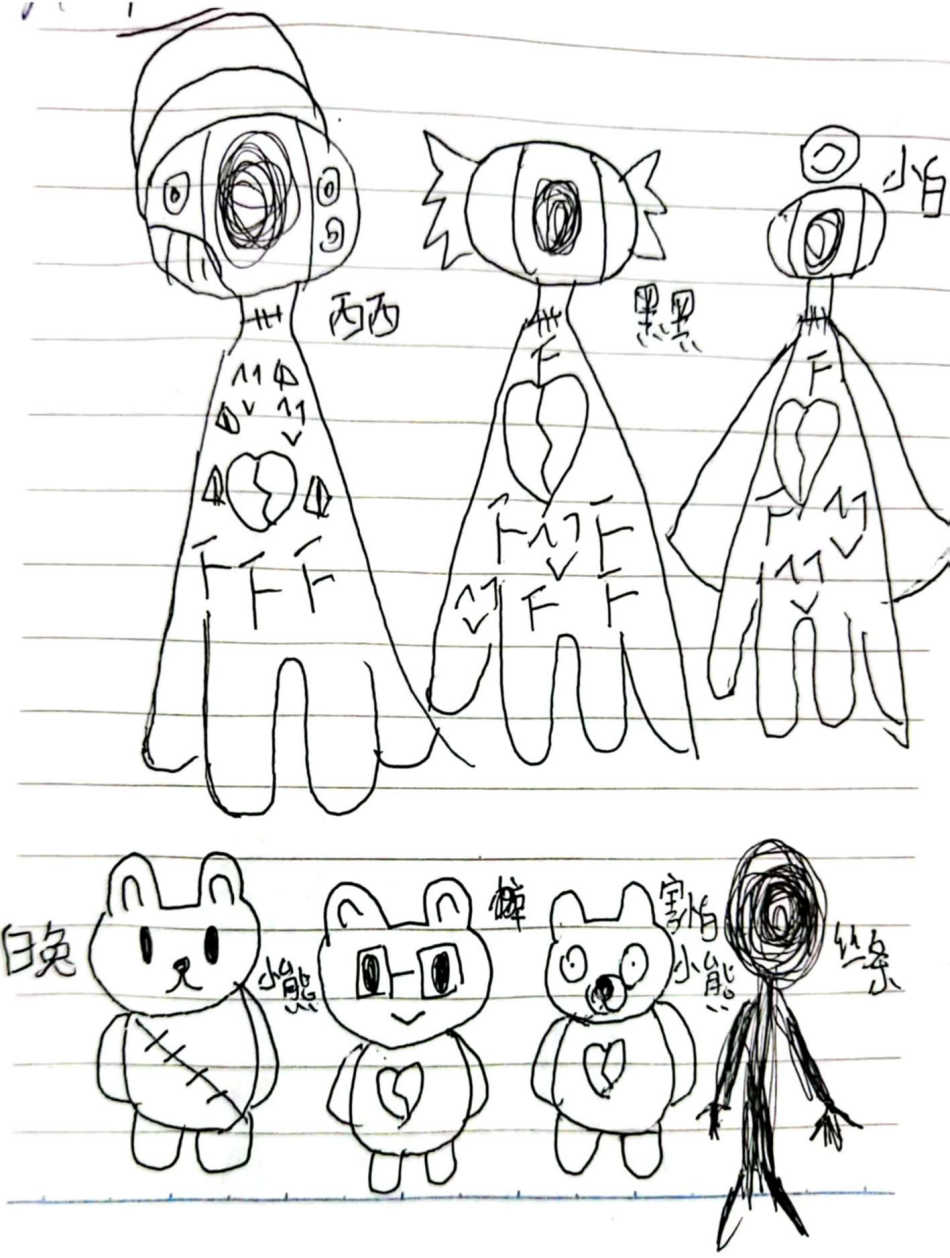
The patient described that there were different identities within her, which she named Xixi, Heihei and Xiaobai. When others noticed her feeling sad and distressed, she said she was Xixi. When others noticed her emotional lability and irritability, she stated that she was Heihei. When others observed her state of calmness, she stated that she was Xiaobai. She also mentioned the presence of various little monsters inside her, which she called “White Rabbit”, “Little Bear” and “Scared Little Bear”. She compared her depressive feelings to tangled threads, which caused her intense distress.

A 15-year-old female was admitted to the hospital with the chief complaints of recurrent self-harm and auditory hallucinations lasting for two months. After admission, the doctors conducted a detailed psychiatric examination on the patient. From February 2025, she began experiencing depressed mood, loss of interest, fatigue, self-blame and guilt, impaired concentration, memory deficits, tremors, headaches, insomnia, irritability, feelings of meaninglessness, suicidal ideation, and recurrent self-harm behaviors. She experienced visual hallucinations, seeing multicolored figures and ghosts out of nowhere, sometimes on the ceiling, sometimes on the windows. She also had auditory hallucinations and heard the voices of boys and girls speaking to her. She believed she deserved punishment and felt pervasive worthlessness. She experienced command auditory hallucinations from a male voice telling her to die and that no one loved her. She engaged in self-harming behavior, using a fruit knife to cut her wrists, which showed multiple superficial cuts. She expressed negative thoughts, stating that she was a burden on her family and had no value, and expressed suicidal ideation. She also presented with dissociative symptoms, feeling like an empty shell and perceiving the surrounding world as unreal. Her supervising psychiatrist and nurse observed that the young girl presented with three distinct identities. Each identity had a defined function. Switching among these three identities occurred in response to varying situations. To gain a deeper understanding of her inner world and characterize her various identities, the supervising psychiatrist recommended depicting these personality states through drawing. She drew a variety of different identity states, named them, and explained their conditions (**Figure 1**).

The patient stated that she was repeatedly bullied by her classmates during elementary school. She informed her parents and teachers, but these concerns were not addressed, and no assistance was provided to resolve the issues. She is the eldest of four siblings (having two younger sisters and one younger brother). Her mother was the primary caregiver; her father worked away from home as a migrant laborer. She described her father as prone to anger, frequently subjecting her to verbal abuse and occasional physical abuse. Consequently, she became unwilling to communicate with her parents about problems, feeling they would not support her. This situation eventually progressed to depressed mood, school phobia, and an unwillingness to attend school, resulting in her withdrawal from school.

Her mother had a history of postpartum depression and achieved clinical stability following treatment. At present, her mother has discontinued the medication and resumed normal functioning. Her maternal grandfather had a history of depression and was once admitted to a psychiatric hospital. He passed away due to complications from myocarditis and pneumonia.

## 3 Diagnostic assessment and treatment

**Figure 2:**
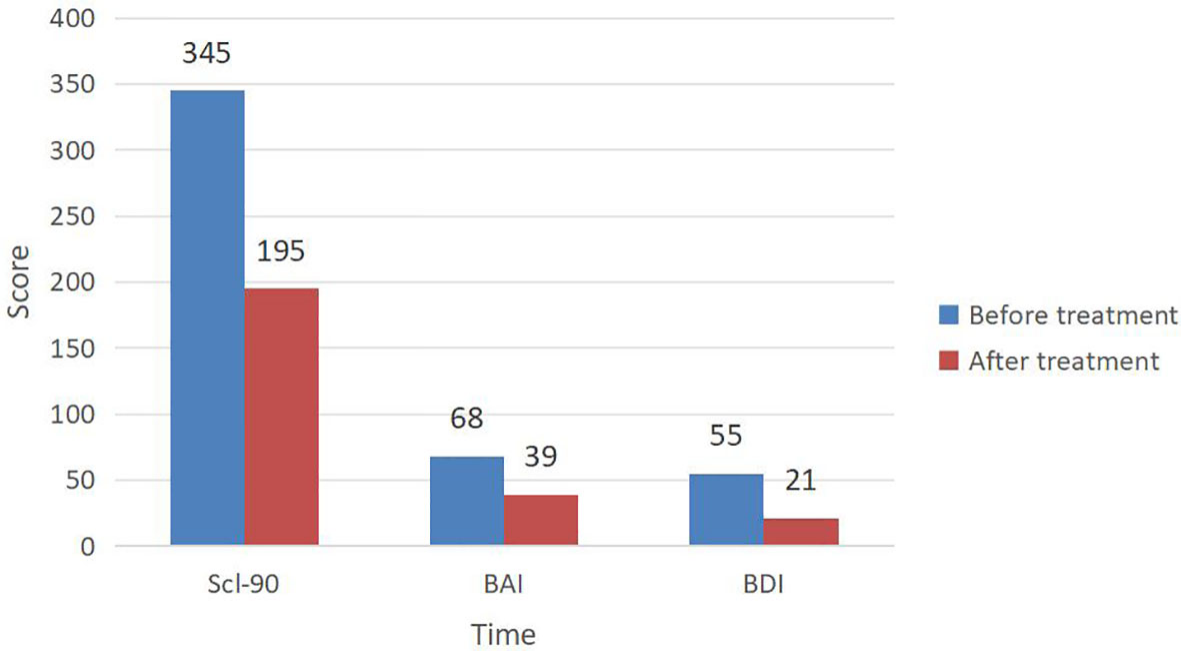
Changes in total scores of each scale before and after treatment.

According to DSM-5 criteria, she was diagnosed with Dissociative Identity Disorder and Major Depressive Episode, Severe, With Psychotic Features. The patient exhibited severe suicidal ideation and engaged in self-harming behavior manifested as biting and scratching of the arms. After admission, she was assessed using the Symptom Checklist 90 (SCL-90), Beck Anxiety Inventory (BAI) and Beck Depression Inventory (BDI) with total scores of 345, 68 and 55, respectively (**Figure 2**).

Prior to her diagnosis with dissociative identity disorder and depression, her psychotherapist conducted several sessions of psychotherapy to help her cope with past trauma, focusing primarily on stabilization techniques; however, due to her severe suicidal ideation, self-harming behavior and auditory hallucinations, the psychotherapy had little effect. To alleviate depressive symptoms and psychotic symptoms, sertraline and olanzapine were administered in combination therapy, with doses gradually increased. The maximum dose of sertraline was 200 mg/day, and that of olanzapine was 15 mg/day. However, the patient still had negative thoughts and repeated behaviors of harming herself. The attending physician recommended combined ECT, and the patient and her guardian agreed to this treatment plan. The attending physician obtained written informed consent from the patient and her guardian. During the informed consent process, the attending physician informed the patient and her guardian about the possible adverse effects of ECT, such as headache and reversible memory loss. The patient received ECT with a Thymatron device (Somatics, USA). Before each treatment session, the patient fasted for a minimum of 8 hours, vital signs were measured, and the bladder was emptied. Pre - treatment medications consisted of succinylcholine chloride (20 mg) as a muscle relaxant, atropine (0.5 mg) to decrease airway secretions, and etomidate (12 mg) for anesthesia maintenance. The electrodes were bilaterally placed on the patient’s temporal lobes with a pulse width 1 ms. The initial stimulus dose was calculated as two - thirds of the patient’s age, and subsequent doses were adjusted according to the patient’s physical condition and seizure responsiveness.

**Figure 3:**
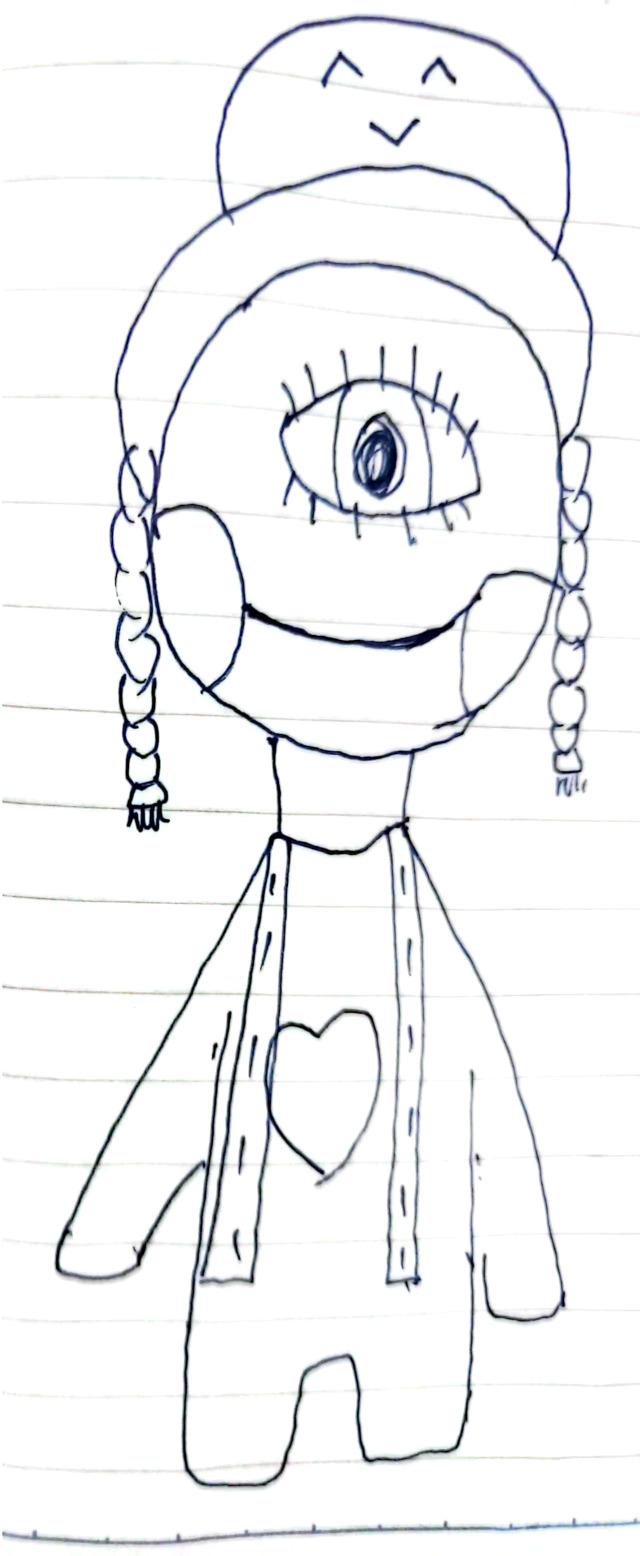
After receiving two sessions of ECT, her identities achieved integration, “the little monster” vanished, her suffering alleviated, and she began to feel a sense of joy.

Following two sessions of ECT treatment, the patient showed significant improvement in symptoms of DID. She no longer reported the existence of multiple distinct identities. This change was reflected in her drawings, and she reported an improved mood (**Figure 3**). However, persistent auditory hallucinations and negative thoughts remained. She specifically believed substantial money had been spent on her care and erroneously perceived her death as a way to reduce the family’s financial burden. Treatment continued with a combination of ECT and pharmacotherapy to alleviate her depressive and psychotic symptoms.

Following a course of 12 sessions of ECT (**Table 1**) in conjunction with pharmacotherapy, the patient demonstrated significant clinical improvement in DID symptoms. During the course of ECT, adverse effects observed included mild headache and transient memory impairment.

**Table 1:** The patient’s ECT treatment history.

Time	MECT	Charge(100%=504mC)	Pulse width(millisecond, ms)	Seizure duration(second, s)
March 21,2025	First time	10%	1ms	32s
March 22,2025	Second time	10%	1ms	47s
March 24,2025	Third time	15%	1ms	20s
March 26,2025	Fourth time	20%	1ms	42s
March 28,2025	Fifth time	20%	1ms	42s
March 31,2025	Sixth time	20%	1ms	37s
April 7,2025	Seventh time	20%	1ms	45s
April 11,2025	Eighth time	20%	1ms	57s
April 14,2025	Ninth time	20%	1ms	46s
April 16,2025	Tenth time	20%	1ms	53s
April 18,2025	Eleventh time	20%	1ms	33s
April 21,2025	Twelfth time	20%	1ms	38s

After 36 days of treatment, the patient reported improvement in depressive symptoms, denied suicidal ideation, and asked to be discharged from the hospital. Following a comprehensive risk assessment, discharge was arranged. Prior to discharge, a symptom assessment was performed utilizing the SCL-90, BAI and BDI with the scores of 195, 39 and 21, respectively (**Figure 2**). Following treatment, the patient demonstrated significant reductions in scores on SCL-90, BAI and BDI compared to baseline (**Figure 2**).

## 4 Discussion

### 4.1 DID and trauma

The trauma model of dissociative identity disorder (DID) posits that this disorder stems from chronic neglect, physical abuse, and/or sexual abuse in childhood (8). The Australian case series involving 62 cases of dissociative identity disorder (DID) (15), as well as this current case report, provides support for the trauma model. In Australian case reports, 87% of the patients provided a history of sexual abuse in their childhood. This case report reveals that the patient suffered from chronic emotional neglect and physical abuse during childhood. Her mother suffered from depression, and with four children to care for in the family, her emotional needs were unmet. Her father subjected her to physical abuse. The above causes may contribute to the development of DID in this patient.

### 4.2 DID and comorbidities

Nearly all patients with DID have comorbid psychiatric disorders, the most prevalent being depression, PTSD, borderline personality disorder, self-injurious behaviors and substance abuse (16). As a result, their clinical presentations are complicated, making the condition liable to be missed or misdiagnosed. It is estimated that approximately 1.5% of the world’s population suffers from dissociative identity disorder, with females having a higher prevalence than males, with a ratio of females to males of 1.75 - 1.9:1 (16). It takes an average of 5-12 years from the first contact with mental health services for a patient to receive a correct diagnosis (17). This condition is frequently misdiagnosed as depression, anxiety, or borderline personality disorder (18). The patient in this case report was misdiagnosed with schizophrenia at the local hospital. The patient’s symptoms did not improve with the use of antipsychotics. She was admitted by her family to a tertiary teaching psychiatric hospital for further assessment and treatment. The doctor conducted a thorough physical and psychological examination, and medical staff held regular interviews with her. Based on the diagnostic criteria of the DSM-5, she was diagnosed with dissociative identity disorder with comorbid depression. Severe depression and self-injurious behaviors noted in this case align with the high Axis I and II comorbidity rates of DID reported in the literature (16). Differentiation can be made from psychotic disorders: patients with DID frequently exhibit hallucinatory symptoms, yet they have better cognitive insight than individuals with schizophrenia and lack true delusions (16).

### 4.3 Treatment of DID

The treatment of DID includes medication, psychotherapy, and physical therapy. At present, there is no specific medication, and most treatments are symptomatic. Given the significant emotional comorbidity, marked functional impairment, and treatment resistance associated with DID, identifying potentially useful novel psychopharmacological combinations is crucial (19). Atypical antipsychotics that modulate the 5-hydroxytryptamine 2A receptor and dopamine D2 receptor can be used to treat complex trauma cases with psychotic and intrusive symptoms, and may also be considered in the treatment of DID (20). Selective serotonin reuptake inhibitors (SSRIs) are also effective in treating emotional comorbidities associated with dissociative identity disorder (19, 21). Combination of antidepressants and antipsychotics can improve DID. Two reports indicate that the combination of antipsychotic and antidepressant medications effectively improves dissociative personality disorder and emotional and behavioral problems in adolescents, with stable outcomes maintained at the 6-month follow-up (22, 23). One report involved mirtazapine combined with risperidone, and the other involved fluoxetine combined with quetiapine. In this report, we used sertraline in combination with olanzapine.

ECT has a definite effect on the suicidal ideation of patients with DID comorbid with depressive disorder, but whether it can promote the integration of dissociated personalities still needs further research (14). Of the four patients with DID co-morbid major depression reported by DeBattista et al. (13), three showed significant improvement in depressive symptoms (decrease in Hamilton Depression Inventory scores) after receiving ECT and did not experience an exacerbation of DID symptoms. One of these patients maintained remission after eight weeks of treatment. Four patients were women aged 31–48 years who exhibited severe self-harm or suicidal behavior. All had previously undergone multiple drug treatments (including various antidepressants, mood stabilizers, antipsychotics, etc.), but with limited efficacy. This case report describes a 15-year-old adolescent female who, following treatment with ECT combined with antidepressant and antipsychotic medication, experienced improvement in depressive symptoms and integration of distinct personality states. However, it has also been reported that ECT may induce transient dissociative DID symptoms in depressed patients with a history of childhood trauma (24). Neuroimaging studies suggest that DID patients exhibit functional abnormalities in the orbitofrontal cortex and cortico-limbic systems (16). ECT may rapidly stabilize patients’ crisis symptoms by modulating these neural networks associated with emotion regulation and self-awareness (16). Regarding ECT parameters, we acknowledge that unilateral right stimulation and 0.5ms pulse width is the preferred standard for minimizing cognitive side effects. Given that the patient was exhibiting life-threatening symptoms and required a rapid response, we opted for bilateral temporal stimulation and a pulse width of 1.0 ms; however, we are aware that this deviates from current best practices aimed at minimizing cognitive side effects. When treating such complex cases, clinicians should carefully weigh these factors and, when conditions permit, conduct prospective monitoring of cognitive function.

A particularly noteworthy aspect of this case is the patient’s mixed response to ECT: after only two sessions, the symptoms of marked personality changes had already subsided, whereas the auditory hallucinations and delusions of guilt required further treatment. This finding contradicts the existing literature, which generally supports the efficacy of ECT in treating auditory hallucinations and delusions associated with depression (25). We hypothesize that this discrepancy stems from factors potentially related to the unique nature of “psychotic” symptoms in DID. Auditory hallucinations in individuals with DID originate internally (26) and may be associated with higher levels of trauma and dissociation. The delusional features of dissociative identity disorder primarily manifest as distrustful beliefs stemming from traumatic childhood experiences, and these delusions are closely associated with dissociative symptoms such as depersonalization and derealization (27). Once the acute phase has been managed, psychological therapy for the trauma is necessary.

Expert consensus guidelines recommend long-term psychotherapy as the primary treatment for DID (28). Once her condition had stabilized, the psychotherapist provided her with psychoeducation on DID, and taught her specific skills to maintain a sense of safety, improve emotional tolerance, manage dissociative symptoms using grounding techniques, and cope with intrusive post-traumatic thoughts using containment techniques.

## 5 Conclusion

For adolescent patients with DID comorbid with depression who are at high risk of suicide, ECT combined with psychiatric medication may be considered a relatively effective and safe treatment strategy in emergency situations. However, the duration of the treatment effect and whether it is effective in other adolescent patients is not known. Studies with larger samples are needed to observe the maintenance of efficacy and long-term adverse effects.

## Patient perspective

Following a ten - month follow - up period, the patient has consistently and strictly adhered to her prescribed regimen of sertraline and olanzapine. She underwent regular psychotherapy. Her family showed her more care. She continues to report occasional episodes of low mood and remains on academic leave. However, her memory function has significantly improved, with no recurrence of hallucinations or depersonalization symptoms. She no longer experiences suicidal thoughts or self - harming behaviors and feels connected to reality. She is now able to handle daily household tasks and engage in normal social interactions with her peers. ECT reduced her risk of suicide during the acute phase, but comprehensive treatment facilitated her recovery.

## Data Availability

The raw data supporting the conclusions of this article will be made available by the authors, without undue reservation.
